# Threshold concepts in health professions education research: a scoping review

**DOI:** 10.1007/s10459-022-10127-5

**Published:** 2022-06-16

**Authors:** Matilda Liljedahl, Per J. Palmgren, Cormac McGrath

**Affiliations:** 1 Department of Oncology, The Institute of Clinical Sciences, Sahlgrenska Academy at the University of Gothenburg, Box 426, 405 30 Gothenburg, Sweden; 2 Department of Oncology, Sahlgrenska University Hospital, Gothenburg, Sweden; 3 Department of Learning, Informatics, Management and Ethics, Karolinska Institutet, Stockholm, Sweden; 4 Department of Education, Stockholm University, Stockholm, Sweden

**Keywords:** Clinical education, Health professions education, Medical education, Research methods, Scoping review, Threshold concepts

## Abstract

Threshold concepts (TCs) are increasingly used in health professions education (HPE) research. TCs are claimed to be conceptual gateways which are often traversed with substantial difficulty. In this paper, we report on a scoping review investigating the following research question: What is the scope and nature of the currently available research on threshold concepts in health professions education literature? We employed Arksey and O’Malley’s model for scoping reviews. A search for literature on TCs in HPE research between 2003 and 2020 yielded 999 records of which 59 were included in the review. The data set was subject to quantitative descriptive analysis of article characteristics as well as qualitative thematic analysis of the scope of research on TCs. Among the 59 articles selected for review, there were 30 empirical, 26 conceptual and three reviews. A majority were published in 2015 or later. Almost half of the included articles attempted to identify possible TCs within HPE. Others investigated how TCs can be traversed or suggested how TCs could influence curriculum design. Some critically appraised the framework of TC. Although TCs are increasingly utilised in HPE, the present review identified how researchers came across methodological challenges related to identifying possible TCs and definitional challenges around identifying the essential characteristics of TCs. Before embracing TCs as the next go-to theory for learning in HPE, we acknowledge the need for methodological stringeny and rigour as well as more data to support TCs. Until then, any implementation of TCs in HPE curricula should be done cautiously.

**Supplementary Information:**

The online version contains supplementary material available at 10.1007/s10459-022-10127-5.

## Introduction

Threshold concepts (TCs) have recently gained broader acceptance and traction in health professions education (HPE) research and there are now a substantial number of research publications exploring TCs both conceptually and empirically (Gaunt & Loffman, 2018; Neve et al., 2016). It is argued that TCs hold the potential to innovate teaching and learning in HPE (Amin, 2019) as they challenge traditional ways of educational practice; however they can also be difficult to comprehend (Neve et al., 2016). At their core, TCs build on the idea that some knowledge is of a particularly troublesome nature and inhibits learning of other related knowledge (Perkins, 2006). TCs first emerged from the *Enhancing Teaching–Learning Environments in Undergraduate Courses* project launched in the UK, where Meyer and Land further developed the idea of troublesome knowledge and postulated that:

“A threshold concept can be considered as akin to a portal, opening up a new and previously inaccessible way of thinking about something. It represents a transformed way of understanding, or interpreting, or viewing something without which the learner cannot progress. As a consequence of comprehending a threshold concept there may thus be a transformed internal view of subject matter, subject landscape, or even world view”.(Meyer & Land, 2003, p. 412)

Importantly, Meyer and Land distinguish TCs from the idea of core concepts, arguing that core concepts need to be understood as conceptual ‘building blocks’, but do not necessarily alter one’s world view (Meyer & Land, 2003). Meyer and Land presented TCs as an evolving conceptual framework (Meyer & Land, 2005) and as the notion of TCs has become more widespread, several specific features have been identified (Baillie et al., 2013; Meyer & Land, 2005). TCs are said to be *transformative* in that, once they are understood, a TC is expected to change the way learners view their discipline (Meyer & Land, 2003). The change that results from comprehending a TC is, according to Meyer and Land (2003), both powerful and compelling. Moreover, it is argued that understanding a TC can alter how learners think about their field, about themselves, or about the world. TCs are *troublesome* in that the concepts in question may be perceived by learners as counterintuitive, or seemingly incoherent (Meyer & Land, 2005). The troublesomeness addressed in TCs is closely aligned with and related to the work of Perkins (1999), who previously explored the idea of troublesome knowledge. Meyer and Land (2003) argue that TCs are troublesome in the sense that they are onerous for learners to understand. TCs are *irreversible* in that once a TC is understood, learners are unlikely to forget it (Meyer & Land, 2003). By way of explanation, it may be difficult for teachers to retrace the journey back to their own days of ‘innocence’, before they had crossed the TC portal (Meyer & Land, 2005). TCs are *integrative* in that mastery of a TC often allows learners to make connections between disparate concepts and their scope that were previously veiled, and it may also facilitate the integration of different aspects of a subject or discipline (Meyer & Land, 2003). Consequently, TCs expose the hidden interrelatedness of a phenomenon. Furthermore, TCs are *bounded* and delineate a particular conceptual space, serving a specific and limited purpose (Meyer & Land, 2003). TCs are *discursive*, thus traversing a TC is likely to empower learners to incorporate an enhanced and extended use of language to express, contemplate, and convey learned ideas (Meyer & Land, 2005). TCs are *reconstitutional*, meaning that they reconstruct existing knowledge in a way which causes an ontological shift in an individual’s understanding. Lastly, TCs are *liminal* in that they involve a process or journey where understanding, misunderstanding and confusion are states in which a learner will pass before transformation (Meyer & Land, 2005).

Critics of TCs argue, among other things, that TCs have major definitional concerns and empirical shortcomings. Some argue that TCs are portrayed in different and sometimes incompatible ways, and that there is insufficient empirical consensus on what constitutes a TC (Nicola-Richmond et al., 2018; Rowbottom, 2007; Salwén, 2019). Rowbottom argues that, given the original authors’ inability or unwillingness to clearly identify what a TC is, it is surprising that other scholars have been so willing to identify TCs in their own contexts (Rowbottom, 2007). Recently, three areas of concern relating to TCs have been raised in the field of HPE research (Brown et al., 2021). The first concern relates to demarcation and definitional problems, where it is argued that TCs lack a coherent and clear definition. The second concern relates to the body of knowledge problem and issues associated with identifying a singular body of knowledge encapsulated by a TC. The third concern relates to TCs being articulated as component parts of professional identity formation, in that such explanations lack grounding in the literature (Brown et al., 2021). We acknowledge that there is an emergent conceptual, empirical, and critical literature on the utilisation, status and merits of TCs in HPE research. The available reviews and syntheses have provided some insights into the literature on TCs with regards to specific aspects or fields in HPE, such as how TC can inform curriculum design (Barradell & Peseta, 2017) and TCs’ potential usefulness in medicine and surgery (Amin, 2019). However, we identify a need to map the available research literature in the field of HPE based on a systematic search. Consequently, to better understand how TCs have been used and understood in HPE research, a scoping review was undertaken, posing the question: What is the scope and nature of the currently available research on threshold concepts in the health professions education literature?

## Methods

A scoping review is a way of mapping the research literature on a given topic and to identify existing research gaps (Arksey & O'Malley, 2005; Levac et al., 2010). Scoping reviews involve an iterative process and allow for the inclusion of a broad range of scientific literature emerging from various research designs (Khalil et al., 2016). This scoping review is based on the five-stage review methodology framework outlined by Arksey and O’Malley (2005). The Preferred Reporting Items for Systematic Reviews and Meta-Analysis extension for Scoping Reviews (PRIMSA-ScR) Checklist was employed and is added as online Appendix 1.

### Stage 1: identifying the research question

After reviewing the literature on TCs and other closely related concepts such as ‘transformative learning’ and ‘troublesome knowledge’, the research team appraised that ‘threshold concept’ was a specific and unique concept that could be used in an extensive search. The following research question was posed: *What is the scope and nature of the currently available research on threshold concepts in the health professions education literature?* Drawing on the work of Arksey and O’Malley (2005) to achieve a broad scope, first, the extent, range and nature of research activity were examined, and second, research gaps in the literature were identified. In line with the broad scope of the review, it was decided to include learners at all levels of HPE, such as undergraduate and postgraduate education.

### Stage 2: identifying relevant studies

After fine-tuning the research question, a preliminary and broad search in a variety of search engines was initiated to find any existing reviews on the topic. The search was limited to articles written in English and published between 2003 and 2020. 2003 was chosen as a point of departure as Meyer and Land’s seminal article was published that year. Two search engine experts (university librarians) conducted searches on November 3, 2020, in what we identify as three central databases for HPE researchers: Medline (Ovid), Web of Science Core Collection (Clarivate) and CINAHL (EBSCO). Thesaurus terms were used for database-specific keywords by using combinations of the following truncations: “threshold* concept*” in title, abstract and/or keywords. The documentation of search strategies can be found in online Appendix 2. A supplementary literature search was performed on March 25, 2021, to identify late and deferred publications in 2020. No hand-searching of specific journals were performed.

### Stage 3: study selection

**Fig. 1:**
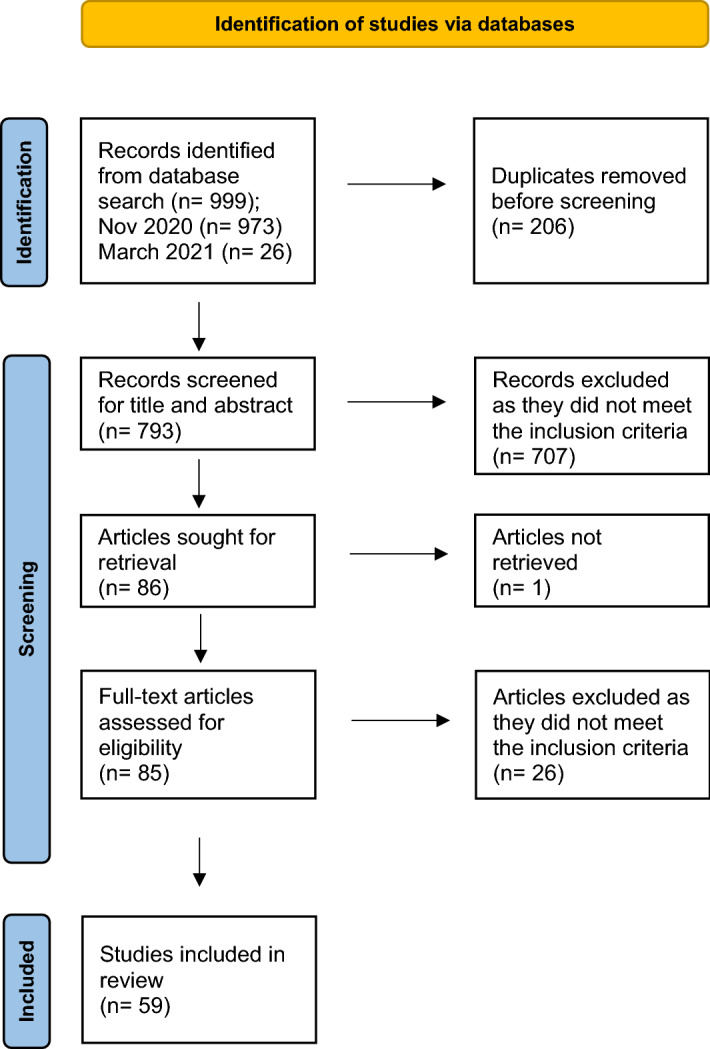
PRISMA flow chart illustrating the identification and selection of studies for review

After duplicates were identified and removed, all search records were imported to a reference management software ('Mendeley Reference Manager,' 2021). The titles and abstracts of all records were screened by one of the three authors using the following inclusion criteria: (1) related to TCs, (2) addressed learners within HPE including undergraduate and postgraduate education and learning among professionals, (3) published in scientific peer-reviewed journals, (4) not mainly an overview of TCs. In the review process, no articles were excluded based on methodology or type of article. Commentaries and letters to the editor were included, but not theses or conference papers. After a first screening, borderline cases were screened again and discussed by the research team until consensus was reached. Subsequently, full texts were screened resulting in the exclusion of records that did not comply with the inclusion criteria. After duplicates had been removed, a total of 793 records were identified on the initial and supplementary search, from which ultimately 59 articles were selected for further analysis. The majority of excluded articles were removed as they did not pertain to TCs. Other reasons for exclusion were; articles investigating learners within other fields then HPE, not published in peer review journals or because it was a conference paper. The Preferred Reporting Items for Systematic Reviews and Meta-Analysis (PRISMA) guidelines (Page et al., 2021) were used to report the flow of the included articles in this review (see Fig. 1).

### Stage 4: charting the data

Relevant demographic data from the 59 articles were extracted, condensed and entered into a data-charting spreadsheet. The articles were given identification codes and the data charted included information about the authors, title, year of publication, journal, health care profession, study location, study design, data collection method, primary focus of study (aim and/or research questions), the articles referred to and how TCs were applied. After the first iterative round, details in the spreadsheet were revised to include all articles in the data set. Articles using some form of original data (review articles excluded) were categorised as *empirical* and those articles addressing or building on theory, literature, opinions, and/or experiences were classified as *conceptual*. The conceptual data thus included both theoretical work and commentaries as it was difficult to make a clear distinction between them, not least because different journals assort types of articles differently. All charted data were then cross-checked to establish consistency in the process and to harmonise semantic differences between the authors.

### Stage 5: collating, summarising and reporting results

In the last stage, data were analysed in two distinct ways: (1) through a quantitative descriptive analysis providing insight into the extent, nature and distribution of the included studies, and (2) through a qualitative thematic analysis informed by the research question (Levac et al., 2010); What is the scope and nature of the currently available research on threshold concepts in the health professions education literature? For the qualitative thematic analysis, we randomly selected 20 articles, using an internet-based randomisation generator. We commonly reviewed five of the articles and additionally, each author reviewed five articles which was only reviewed by that author. In the subsequent debriefing session, therefore, 1/3 of the data set were scrutinised one by one, discussing the scope, findings and conclusions of each paper. Based on this reading and debriefing, three preliminary themes on the scope and nature of available research were identified. The first author then re-examined the entire data set to confirm the preliminary themes and to search for additional ones. Going back and fourth between our data set and the preliminary themes as well as debriefing and discussing borderline cases with all authors, we jointly arrived on four themes to describe HPE research on TCs.

### Research team and reflexivity

The research team comprised experienced researchers within HPE. We had come across TCs in our research and as faculty developers, but none of us had researched TCs previously. As many others, we found TCs to be appealing but also noted the more critical literature on the subject. We wanted to grasp the scientific discussion about TCs and hence decided to conduct a scoping review. Performing the review was a highly iterative and collaborative activity as we were all very much involved in every step of the research process. This approach enabled us to communicate on methodological choices and to converse emerging understandings of the literature we reviewed (Thomas et al., 2020).

## Results

A total of 59 articles were selected to examine the scope and nature of the currently available research on TCs in the HPE literature. The following section contains first a descriptive analysis of the articles and then the four themes generated from the thematic analysis.

### Descriptive analysis

Of the 59 articles included in the review, 30 (51%) were empirical, 26 (44%) conceptual and three (5%) were reviews. Of the empirical articles, 23 (76%) were qualitative, two (7%) were quantitative, three (10%) used a mixed-method approach, and two (7%) described educational development initiatives.

The data collection methods used in the empirical articles were mainly qualitative individual interviews (n = 12), questionnaires (n = 10), focus group interviews (n = 8), texts (n = 5), or a combination of these. There were also examples of field observations (n = 1), Delphi method (n = 1), and nominal group technique (n = 1). The empirical work included data from the UK (n = 12), Australia (n = 5), the USA (n = 4), Canada (n = 4), New Zealand (n = 3), China (n = 1), Sweden (n = 1), Switzerland (n = 1), and Taiwan (n = 1). Two studies collected data in two countries. The authors of the conceptual work and reviews were, with few exceptions, based in Australia, Canada, New Zealand, UK or USA.

All levels of HPE were represented in the articles, with the majority at the undergraduate level (n = 44). Nine articles referred to postgraduate education, two concerned health care professionals, and in five the level of education was not explicitly stated.

**Fig. 2:**
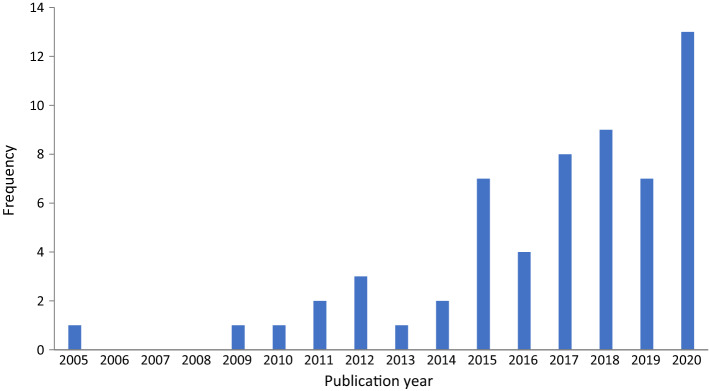
Year of publication. The figure shows that most articles included in the review were published in 2015 or later. The number of articles related to Threshold Concepts in health professions education research shows an upward trend

Literature concerning seven different health care professions was found: medicine (n = 18), occupational therapy (n = 10), nursing (n = 8), dentistry (n = 4), physiotherapy (n = 2), pharmacy (n = 1) and midwifery (n = 1). Fifteen articles were multiprofessional or interprofessional. A total of 31 articles were published in HPE journals, 21 were published in general medicine or health journals, and seven were found in higher education journals. The large majority (n = 48) were published in 2015 or later (Fig. 2).

### Qualitative thematic analysis

The qualitative thematic analysis of the main scope of TC research in HPE revealed four major themes (see Table 1): *Identifying TCs, Investigating how TCs can be traversed, TCs influencing curriculum design*, and *Critically appraising TCs*. Many articles primarily situated in one theme also included another theme and thus engaged with TCs with more than one scope.

**Table 1:** An overview of the four themes

Theme	Description	Included articles
Identifying TCs	Attempt to identify TCs in a given area, concept, field, programme or profession	Evgeniou et al., (2015), Moeller and Fawns (2018), Neve et al., (2017), Barradell and Peseta (2016), Bhat et al., (2018), Nicola-Richmond et al., (2016), Tanner (2011), Blackburn and Nestel (2014), Bowman (2017), Green and Rasmussen (2018), Hill (2017), Kolar and Janke (2019), Morgan et al., (2019), O'Callaghan et al., (2020), Barron et al., (2020), Khatri et al., (2020), Leidl (2018), Neve et al., (2020), Clouder (2005), Fortune and Kennedy-Jones (2014), Howarth et al., (2018), Kempenaar and Shanmugam (2018), Larson (2020), Stacey and Stickley (2012), Khurshid et al., (2020), Wearn et al., (2020)
Investigating how TCs can be traversed	Explore or evaluate how a TC can be learned, comprehended or crossed	Fredholm et al., (2020), Kaelin and Dancza (2019), Vaughan (2016), Rodger et al., (2015), Stacey and Pearson (2018), Stacey et al., (2015), Ho et al., (2020), Chen et al., (2020), Nicola-Richmond et al., (2019)
TCs influencing curriculum design	Discussing the way in which TCs could or should influence current curricula of HPE	Angell and Taylor (2013), Barradell (2017), Evgeniou and Loizou (2012), Kinchin et al., (2011), McAllister (2015), McAllister et al., (2015), Nambiar-Greenwood (2010), Neve (2019), Senior and Telford (2015), Hyde et al., (2018), Neve et al., (2016), Levett-Jones et al., (2015), Kneebone (2009), Suibhne (2012), Barry and Littlewood (2017)
Critically appraising TCs	Discussing the nature and conceptualisation of TCs, as well as how they have been, can be or should be researched	Bhat and Goldszmidt (2020), Hill (2020), Chen and Poole (2018), Gupta and Howden (2019), Barradell and Peseta (2016), Crookes et al., (2020), Ma et al., (2017), McBee et al., (2017), Santisteban-Espejo et al., (2020), Barradell and Peseta (2017)

For one article(Barradell & Peseta, 2016), the primary focus could not be decided, so it was situated in two themes, i.e., *Identifying TCs* and *Critically appraising TCs*

#### Identifying TCs

Almost half of the articles tried to identify possible TCs in HPE (n = 26), both in undergraduate and postgraduate education. A vast majority of articles employed qualitative methods to identify TCs (Barradell & Peseta, 2016; Barron et al., 2020; Bhat et al., 2018; Blackburn & Nestel, 2014; Bowman, 2017; Evgeniou et al., 2015; Green & Rasmussen, 2018; Hill, 2017; Kolar & Janke, 2019; Moeller & Fawns, 2018; Morgan et al., 2019; Neve et al., 2017; Nicola-Richmond et al., 2016; O'Callaghan et al., 2020; Tanner, 2011) and a few used mixed methods (Khatri et al., 2020; Leidl, 2018; Neve et al., 2020). Six articles identified TCs from a theoretical perspective, arguing for the validity of TCs on the basis of previous research and educational or theoretical models (Clouder, 2005; Fortune & Kennedy-Jones, 2014; Howarth et al., 2018; Kempenaar & Shanmugam, 2018; Larson, 2020; Stacey & Stickley, 2012). Two articles identified TCs based on literature reviews (Khurshid et al., 2020; Wearn et al., 2020).

Some articles focused on a specific concept or area within a professional field, e.g., microsurgery (Evgeniou et al., 2015), EEG (Moeller & Fawns, 2018), prosthetics (Hill, 2017), palliative medicine (O'Callaghan et al., 2020), prescription writing (Khurshid et al., 2020) and self-directed learning (Bowman, 2017). Other articles identified TCs in a specific area within professions, such as mental health within nursing (Leidl, 2018; Stacey & Stickley, 2012), professionalism among medical students (Neve et al., 2017), internal medicine for residents (Bhat et al., 2018), psychiatry (Khatri et al., 2020), paediatric surgical training (Blackburn & Nestel, 2014), practice education for occupational students (Tanner, 2011), or population health (Neve et al., 2020). Others identified TCs in an entire profession or educational programme, e.g., occupational therapy (Fortune & Kennedy-Jones, 2014; Howarth et al., 2018; Larson, 2020; Nicola-Richmond et al., 2016), physiotherapy (Barradell & Peseta, 2016), dentistry (Green & Rasmussen, 2018), nursing (Stacey & Stickley, 2012) and pharmacy (Kolar & Janke, 2019). Some argued for the existence of TCs in HPE more generally (Clouder, 2005; Kempenaar & Shanmugam, 2018; Wearn et al., 2020), while one focused on interprofessional education (Morgan et al., 2019). A few examples of identified TCs are: (1) Fortune and Kennedy-Jones (2014), who propose how the “relationship between occupation and health” is a TC within occupational therapy; (2) Clouder (2005), who proposes ‘caring’ to be a TC for all health professions education; and (3) Wearn et al. (2020), who conduct a synthesising review exploring ‘professional touch’ through the lens of a TC. Barradell and Peseta (2016) first identified 13 TCs in physiotherapy and later concluded than only one represented a true TC, namely a “client-centred approach and attitude”.

In the empirical work, the articles went about identifying TC in a number of ways. A variety of informants were used, although many used learners such as students e.g., (Bowman, 2017; Green & Rasmussen, 2018) or trainees e.g., (Blackburn & Nestel, 2014; O'Callaghan et al., 2020). Others used clinical educators or educationalists e.g., (Barradell & Peseta, 2016; Tanner, 2011) and some combined several stakeholders as respondents, such as students, educationalists and professionals (Hill, 2017; Khatri et al., 2020) or students, educationalists, and professionals (Nicola-Richmond et al., 2016). In some studies, informants were introduced to TC and then asked to identify potential TCs in their own practice (Barradell & Peseta, 2016; Moeller & Fawns, 2018; Neve et al., 2020). Others did not mention TCs to their informants and instead asked them to describe, for example, difficult concepts, troublesome knowledge or crucial learning experiences (Bhat et al., 2018; Hill, 2017; Leidl, 2018).

#### Investigating how TCs can be traversed

In the second theme, articles were identified that investigated how TCs could be traversed or crossed (n = 9). These articles concerned the process of learning a TC and thus empirically evaluated (Chen et al., 2020; Ho et al., 2020; Nicola-Richmond et al., 2019; Stacey et al., 2015) or explored (Fredholm et al., 2020; Kaelin & Dancza, 2019; Rodger et al., 2015; Stacey & Pearson, 2018; Vaughan, 2016) learners’ experiences. Some introduced a new approach to teaching or learning and evaluated whether or not the new approach stimulated TC learning (Chen et al., 2020; Ho et al., 2020; Stacey et al., 2015). For example, Stacey et al. (2015) introduced co-facilitation as a means to stimulate TC learning, while Chen et al. (2020) evaluated if a blended learning approach could help students overcome difficulties in laboratory courses. Overall, these studies reported favourable findings, meaning that participants felt the TC approach facilitated their learning, although Stacey et al. (2015) pointed out that significant preparation was needed.

Some studies attempted to describe the process of learning a TC. Fredholm et al. (2020), for example, used students’ narratives to describe how they learned TCs in practice, arguing that practical experience can indeed be transformational, impact professional identity development, and be created through practical authentic experiences. Rodger et al. (2015) explored academics’ familiarisation with a TC and used the results to determine the suitability of the TC. Stacey and Pearson (2018) used reflective articles using formative assessment to see how feedback was received, and analysed them from a TC perspective. They concluded that assessment assignments perhaps were not the most suitable data to use when investigating TCs, as TCs involve troublesome knowledge that might be hidden by students in assessment situations.

#### TCs influencing curriculum design

There was also a substantial body of literature describing how curriculum design could or should be influenced by TCs (n = 15). Some introduced TCs to their respective fields and presented it as a promising framework for curriculum reformation and elucidated what distinguished TCs from core competencies or core curricula; Neve et al. (2016) introduced TCs to the field of medical education, Suibhne (2012) to the field of psychiatry and Barry and Littlewood (2017) to the field of anaesthesiology.

Others focused on how the introduction of TCs could reform curricula and proposed it as a novel perspective of learning in HPE. The fields in which TCs are suggested to influence curricula include midwifery students’ learning about infant feeding (Angell & Taylor, 2013), learning to become a physiotherapist (Barradell, 2017), dental education (Hyde et al., 2018; Kinchin et al., 2011), and public health for interprofessional health students (Senior & Telford, 2015).

Some suggested introducing specific innovations into the curriculum as a way to integrate and stimulate learning about the TC, such as films for nursing students to learn recovery practices (McAllister, 2015), scenarios to stimulate interprofessional learning (Nambiar-Greenwood, 2010), and literature to integrate TCs into nursing curricula (McAllister et al., 2015). Neve (2019) argued for how an implementation of TCs into general practice curricula could address the general practitioner recruitment crisis.

The articles incorporated in this theme were all conceptual in nature and the authors provided more or less detailed suggestions for curriculum change. Some used a theoretical and philosophical perspective e.g., (Barradell, 2017; Kneebone, 2009; Neve et al., 2016) to examine how TCs could shift curricular focus from competencies and core knowledge to a transformation in understanding, while others offered more practical insights on how to introduce educational activities to stimulate such learning e.g., (Levett-Jones et al., 2015; McAllister, 2015).

Importantly, many of the articles that focused primarily on identifying TCs within their field (articles found in the theme *Identifying TCs*), also offered detailed suggestions on how these could change existing curricula (Clouder, 2005; Evgeniou et al., 2015; Howarth et al., 2018; Kempenaar & Shanmugam, 2018; Khatri et al., 2020; Larson, 2020; Stacey & Stickley, 2012; Tanner, 2011).

#### Critically appraising TCs

In the fourth theme, the literature critically appraised TCs and how they have been or can be conceptualised and studied (n = 10). In 2017, Barradell and Peseta (2017) published a synthesis of literature on TCs in health professions, including articles from 2003 to 2014. They found the TC literature up until 2014 to be mainly concerned with areas such as complex practices, new conceptualisations of knowledge, and professional identity (Barradell & Peseta, 2017). Hill (2020) was the only researcher to clearly use empirical data to determine what constitutes a TC, when she applied a criteria and concept model to determine whether or not a difficult concept was also a TC. Hill used students and staff to identify five difficult concepts, of which three were also considered to be TCs as they represented both a conceptual and ontological shift. Using the criteria and concept model, Hill (2020) critically appraised the TCs identified by students and staff.

Other work within this theme critically appraised TCs with a basis in their empirical studies or simply from a conceptual perspective in form of, e.g., commentary. Barradell and Peseta (2016) challenged the methodological approach of mainly involving students and educationalists in identifying and developing TCs, arguing that other stakeholders, such as professionals, needed to be involved for TCs to be valid and authentic. Further, they pointed out how the conceptual challenges of TC, for example, if all TC criteria must be fulfilled, may inhibit the way in which students, academics and professionals can engage with TCs (Barradell & Peseta, 2016).

In contrast to the articles primarily focusing on identifying TCs, the articles in this theme were characterised by their concern with the concept itself and elaborating on the very nature of TCs and the relationship between TCs and core concepts or competencies. Bhat and Goldszmidt (2020) discussed whether or not interprofessional education was a TC and concluded that it was, but that it was not possible for education alone to address and enable learning of this TC. Gupta and Howden discussed TCs in the context of longitudinal integrated placements(Gupta & Howden, 2019), while Chen and Poole (2018) questioned the TCs identified by Bhat et al (2018), arguing that some seemed to be true TCs, while others seemed to be skills or skill sets, e.g., “documentation as an essential skill”. There were also examples of literature discussing how TCs could and should be researched. Notably, Crookes et al. (2020) criticised the way in which TCs are currently identified, arguing that a satisfactory method for identifying them is still lacking.

As in the theme *TCs influencing curriculum design* there were articles mainly focusing on identifying TCs that also discussed their nature and how one should go about investigating them (Fortune & Kennedy-Jones, 2014; Hyde et al., 2018; Moeller & Fawns, 2018; Neve et al., 2017, 2020; Nicola-Richmond et al., 2016). Many found the task of identifying TCs to be challenging, which was related to Meyer and Land’s notion that a TC does not necessarily need to fulfil all predefined criteria (Meyer & Land, 2003). Neve et al. (2017) found almost no examples of *boundedness* in their research and hence questioned their identified TCs as TCs. Nicola-Richmond et al. (2016) encountered a similar challenge and decided to consider *transformative* and *integrative* as essential TC characteristics but found, by contrast, only 4 out of 10 of their identified TCs to be *troublesome*. Moeller and Fawns (2018) identified one TC and also two concepts that were understood as *troublesome*, but which did not fulfil all the criteria of a TC. They did, however, not attempt to provide an exhaustive list of TCs, but instead used the TC as a framework for exploring complex skills. Fortune and Kennedy-Jones (2014) argued for a narrow and discipline-bounded conception of TCs, while Hyde et al. (2018) called for greater clarity of TCs and how they relate to other concepts, as well as for the recognition of interindividual differences in experience and mastery of TCs.

## Discussion

This study sought to investigate the scope and nature of the currently avaliable research on TCs in a selection of the HPE literature. Mapping the literature in terms of its volume, nature and characteristics, required a broad approach and a wide selection of research. The findings from this scoping review demonstrate that TCs are increasingly used in both conceptual and empirical research in the broader field of HPE, and that their utilisation involves both opportunities and challenges. The distribution between empirical and conceptual articles was even, and almost half were concerned with identifying TCs within HPE.

The increasing use of TCs is not surprising, as the last 20 years have seen a steady increase in demands for and use of social science theories in HPE research (Laksov et al., 2017; Reeves et al., 2006). As a result, HPE research is increasingly theoretically informed (McGrath et al., 2020; O’Brien & Battista, 2020; Varpio et al., 2020). However, there is also an emerging and critical awareness of how theories and conceptual frameworks are used (Bordage, 2009; McGrath et al., 2020; O’Brien & Battista, 2020; Varpio et al., 2017, 2020). Varpio et al. (2017) identify some of the consequences of the increased use of social science concepts and theories in HPE research, namely, that they may be used without critically reflecting on their ontological and epistemological roots, as well as their definitions and implications. A related concern in this context is that TCs in some cases are being presented with limited formalised or systematic understanding of what constitutes a TC or how it is demarcated, which also is enhanced by Meyer and Land’s (2003) initial non-prescriptive approach. Results from this scoping review suggest that TCs provide an attractive, but seemingly elusive prospect for scaffolding a scientific dialogue on teaching and learning.

Although many articles in this review set out to identify TCs, there was little or no overlap between the TCs that were uncovered, nor were there any studies discovering similar TCs in the same field. Given the number of articles found and the status of TCs in the literature, this is surprising. Consensus is lacking regarding what TCs exist in HPE in general, or in the different disciplines and professions specifically. There is a need for further research focusing on specific concepts identified as potential and conceivable TCs, as that would offer a more robust assurance that TCs, in fact, can be manifested. Hill’s (2020) strategy of employing both a criteria model and a concept model may be useful to researchers attempting to establish whether TCs are a feasible way of conceptualising learning in HPE. Without methodological stringency and substantial evidence for the existence of TCs, it seems difficult to draw any conclusions about their status. In our opinion, individual studies demonstrating the existence of context-bound troublesome knowledge do not constitute or legitimise TCs.

Many of the articles used in this review conceded methodological challenges in their research on how TCs can or should be identified. These challenges, addressed in some of these papers, were related to various aspects of TCs and raised questions such as the following: *Who gets to decide what TCs there are? (*Barradell & Peseta, 2016*) Which informants should be invited and involved in TC identification? (*Barradell & Peseta, 2017*) Can learners articulate the types of troublesome knowledge they are encountering, or are the TCs unknowable until they have overcome them? (*Nicola-Richmond et al., 2019*)* In this regard, what learners perceive as TCs may, in fact, resemble vague symptoms or approximations, and drawing far-reaching conclusions may be unwise. The challenges that investigators possibly encountered when exploring TCs made them call into question the very TC they had identified and construed. While the literature, on the whole, does not provide any clear guidance on this matter, it is apparent that there is a need for further methodological recommendations (Crookes et al., 2020; Hyde et al., 2018).

Our results convey a main concern about the TC discourse in HPE research, thus, pertaining to demarcation and definitional issues, and which elements or characteristics need to be satisfied for a concept to be viewed as a TC. Likewise, the proximity and relationship to other similar notions such as core concepts, and troublesome or difficult knowledge, appeared in several of the examined articles (Bhat & Goldszmidt, 2020; Bhat et al., 2018; Chen & Poole, 2018; Evgeniou et al., 2015; Hill, 2020; Hyde et al., 2018; Moeller & Fawns, 2018; Neve et al., 2017, 2020; Nicola-Richmond et al., 2016, 2019; O'Callaghan et al., 2020; Suibhne, 2012; Tanner, 2011). This concern is not unique to the HPE research community, but echoes literature in other research fields (Barradell, 2013; Rowbottom, 2007; Salwén, 2019). This problem of conceptual ambiguity is mirrored in the recent critical work of Brown et al. (2021), who address ‘The floating signifier problem’, where concepts have varied meaning for different people and there seems to be little agreement on what constitutes a TC. In some of the work in the present review, several of Meyer and Land’s key TC criteria were not deemed as necessary requirements, which prompts the questions: *What, then, is a TC if it does not fulfil the core criteria ? Is it possible to determine the necessary criteria for a concept to be viewed as a TC?* Without consensus on such key aspects, it is difficult to endorse and contribute to an informed scientific dialogue about what really constitutes a TC, when the merit of the concept may be called into question. While we acknowledge that emerging theories are not fixed entities, but continually developed and falsified by modelling and empirical testing, theories become stronger as more data is presented to support that theory (Varpio et al., 2020), but such data is missing in the TC literature reviewed here. This review supports Brown et al.’s (2021) contention that imprecise language is not helpful in taking the HPE field forward. Given the definitional and demarcational concerns, TCs need to be challenged as the next go-to theory for learning for HPE researchers. More work is needed to explore, challenge and fine-tune potential TCs in relation to the original ideas presented by Meyer and Land. Such work is necessary to generate sufficient scientific support for TCs as a promising and worthwhile conceptual framework used in scholarly investigations of HPE. Future research could therefore focus on corroborating potential TCs in other settings and/or from various stakeholders’ perspective, and, more importantly, critically challenge the identified TC anchored in the conceptual literature on TCs. Concerns related to demarcation issues are important, not least due to the enthusiasm in the HPE literature to use TCs as a framework to change curricula. In that regard, it is surprising that so many HPE researchers (found in the theme *TCs influencing curriculum design*) suggested TCs as a novel and suitable model for learning in their field, as a large part of the literature questions the very existence of TCs. In the articles promoting TC as a framework for influencing curricula, definitional challenges were rarely addressed; rather, it seemed to be a pedagogical tool that was taken for granted. While there seems to be value in using the TC as an analytical lens for discussions about learning in HPE, implementing it without reflection upon its definitional challenges and empirical shortcomings seems unwise.

### Limitations

In this review of the HPE literature on TCs, several challenges were encountered. It was decided early in the designing phase of the present study to include literature from basic to clinical science, as well as all professions in health care. Although one could dispute the relevance of including articles primarily focusing on learning in a basic science setting, those articles comprised a small minority of the data set and we would argue had no substantive impact on the findings. Although almost 800 scientific records were screened for this review, it is also possible that some relevant articles were missed. Grey literature such as theses, conference papers and book chapters were not included in the review. Non-peer-reviewed articles, as well as articles not published in English were also discarded. Consequentely, this could have led to an omission of interesting literature from the review. For instance, a thesis by Martindale (2015) was found, which could have been an interesting addition. However, in this paper, we only examined peer-reviewed research publications, as we identified that peer-reviewed publications in the specific databases offers a necessary level of quality. We argue that the chosen approach enabled a broad scoping review of high quality which provided novel insights into the field of HPE but also welcome every attempt to search and map the grey literature on TCs. We did not register any review protocol beforehand. Underway, this has been suggested as a potential improvement for future scoping reviews in HPE (Maggio et al., 2021) and looking back we conclude that this could have increased the transparency of our study.

## Conclusion

Recently, TCs have been put forward as a promising conceptual framework in HPE. This scoping review is, therefore, a much-needed exploration of TCs and reveals an increasing utilisation of TCs in the last couple of years. We conclude that the HPE literature utilising TCs represents a broad range of professions and a broad range of methodologies. Although a substantial body of literature has attempted to define TCs, there remain methodological and definitional challenges that have hindered those attempts. TCs are therefore called into question as the next go-to theory for learning in HPE. Future research should focus on validating identified TCs from other perspectives and in various settings as well as critically challenge TCs in relation to the conceptual literature. We acknowledge the need for methodological stringeny and rigour as well as more data to establish TC as a sound and robust framework. Until then, any implementation of TCs in HPE curricula should be done cautiously.

## Supplementary Information

Below is the link to the electronic supplementary material.

Supplementary file1 (PDF 555 kb)

Supplementary file2 (PDF 207 kb)
