# Supplementary material for: Threshold concepts in health professions education research: a scoping review
Source: Adv Health Sci Educ Theory Pract. 2022 Jun 16;27(5):1457–75. doi: 10.1007/s10459-022-10127-5 (PMC9859919; doi:10.1007/s10459-022-10127-5)
Supplement: Supplementary file 2 — Supplementary file2 (PDF 207 kb) [file 10459_2022_10127_MOESM2_ESM.pdf]

Documentation of search strategies
University Library search consultation group

Date: November 2020

Topic/research question: What is the current available research on threshold concepts in health care professional education?

Name of researcher(s): Per Palmgren

Librarian(s): Emma-Lotta Säätelä & Sabina Gillsund

Databases:

1. Medline (Ovid)
2. Web of Science Core Collection (Clarivate)
3. CINAHL (EBSCO)

Total number of hits:

- Before deduplication: 973
- After deduplication: 767

Comments:

Karolinska Institutet

PRISMA 2009 Flow Diagram[1]

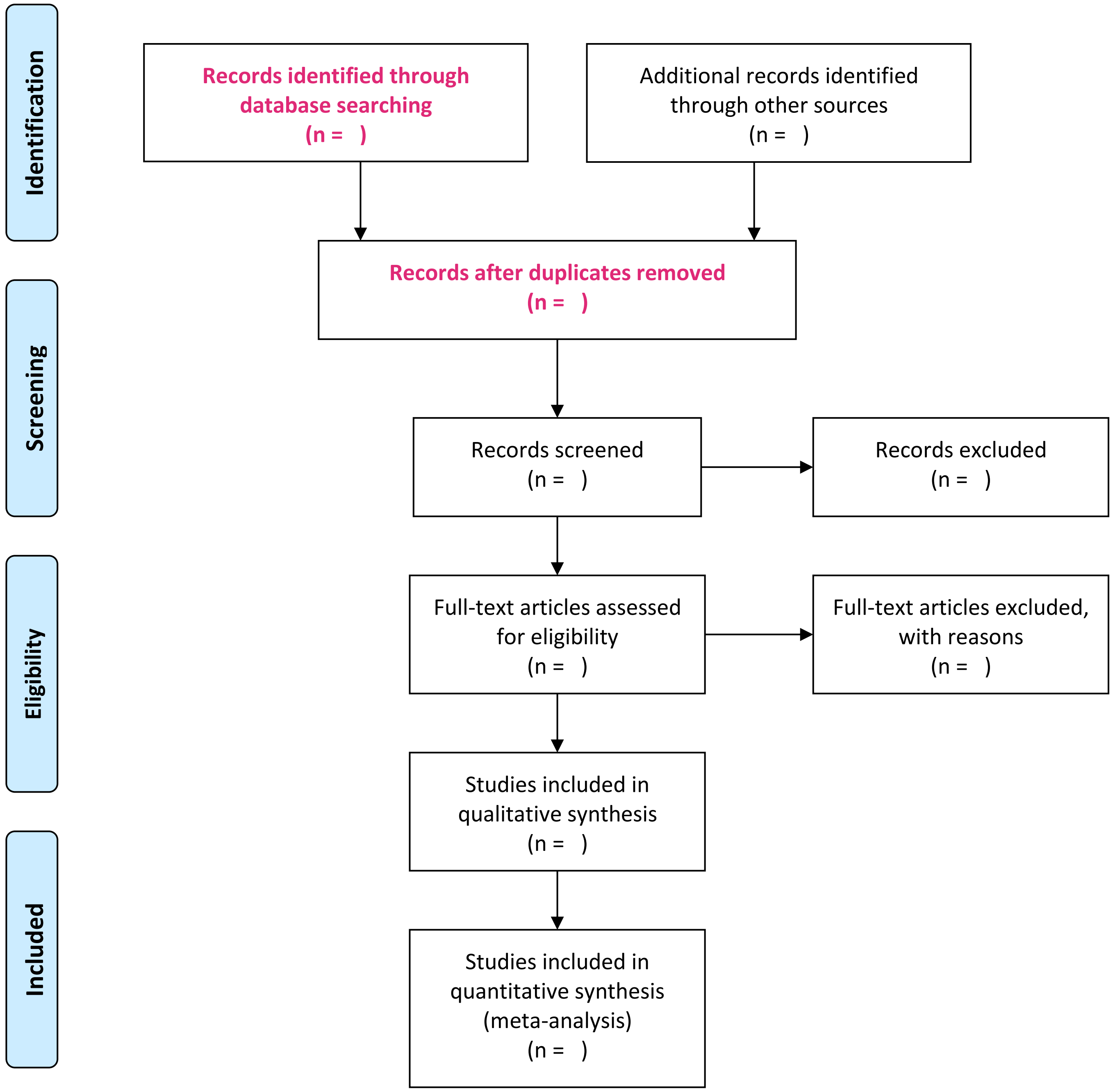

1 From: Moher D, Liberati A, Tetzlaff J, Altman DG, The PRISMA Group (2009). Preferred Reporting Items for Systematic Reviews and Meta-Analyses: The PRISMA Statement. PLoS Med 6(6): e1000097. doi:10.1371/journal.pmed1000097. For more information, visit www.prisma-statement.org.

1. Medline

Interface: Ovid MEDLINE(R) and Epub Ahead of Print, In-Process & Other Non-Indexed Citations and Daily<br><br>Date of Search: 3 November 2020<br><br>Number of hits: 147<br><br>Comment: In Ovid, two or more words are automatically searched as phrases; i.e. no quotation marks are needed	Field labels<br><br>• exp/ = exploded MeSH term<br>• / = non exploded MeSH term<br>• .ti,ab,kf. = title, abstract and author keywords<br>• adjx = within x words, regardless of order<br>• * = truncation of word for alternate endings

Database(s): **Ovid MEDLINE(R) and Epub Ahead of Print, In-Process & Other Non-Indexed Citations and Daily** 1946 to November 02, 2020
Search Strategy:

#	Searches	Results
1	threshold* concept*.ti,ab,kf.	211
2	limit 1 to yr="2003 -Current"	147

2. Web of Science Core Collection

Interface: Clarivate Analytics<br><br>Date of Search: 3 November 2020<br><br>Number of hits: 733	Field labels<br><br>• TS/Topic = title, abstract, author keywords and Keywords Plus<br>• NEAR/x = within x words, regardless of order<br>• * = truncation of word for alternate endings<br><br>Note: sometimes "quotation marks" are needed for single search terms to avoid automatic term mapping (lemmatization).

1 733 **TOPIC:** ("threshold* concept*")
Indexes=SCI-EXPANDED, SSCI, A&HCI, CPCI-S, CPCI-SSH, ESCI Timespan=2003-2020

3. Cinahl

Interface: Ebsco<br><br>Date of Search: 3 November 2020<br><br>Number of hits: 93	Field labels<br><br>• MH+ = exploded Cinahl Heading<br>• MH = non exploded Cinahl Heading<br>• TI = title<br>• AB = abstract<br>• Nx = within x words, regardless of order<br>• * = truncation of word for alternate endings

#	Query	Limiters/Expanders	Results
S1	TI "threshold* concept*" OR AB "threshold* concept*"	Limiters - Published Date: 20030101-20201231	93
